# Effects of exercise of different intensities on withdrawal symptoms among people with substance use disorder: a systematic review and meta-analysis

**DOI:** 10.3389/fphys.2023.1126777

**Published:** 2023-05-10

**Authors:** Hao Li, Wantang Su, Jiajia Cai, Li Zhao, Yan Li

**Affiliations:** ^1^ Department of Exercise Physiology, Beijing Sport University, Beijing, China; ^2^ Key Laboratory of Physical Fitness and Exercise, Ministry of Education, Beijing Sport University, Beijing, China

**Keywords:** exercise, intensity, withdrawal symptom, substance use disorder, meta-analysis

## Abstract

**Background:** Exercise can effectively attenuate withdrawal symptoms and reduce relapse, but it is unknown whether exercise of different intensities produces different results. This study aimed to systematically review the effects of different exercise intensities on withdrawal symptoms among people with substance use disorder (SUD).

**Methods:** Systematic searches for randomized controlled trials (RCTs) on exercise, SUD, and abstinence symptoms were conducted via electronic databases, including PubMed, up to June 2022. Study quality was evaluated using the Cochrane Risk of Bias tool (RoB 2.0) for assessment of risk of bias in randomized trials. The meta-analysis was performed by calculating the standard mean difference (SMD) in outcomes of interventions involving light-, moderate-, and high-intensity exercise for each individual study using Review Manager version 5.3 (RevMan 5.3).

**Results:** In total, 22 RCTs (*n* = 1,537) were included. Overall, exercise interventions had significant effects on withdrawal symptoms, but the effect size varied with exercise intensity and by outcome measure (i.e., for different negative emotions). Light-, moderate-, and high-intensity exercise reduced cravings after the intervention [SMD = −0.71, 95% CI = (−0.90, −0.52)], and there were no statistical differences between the subgroups (*p* > 0.05). Light-, moderate-, and high-intensity exercise reduced depression after the intervention [light, SMD = −0.33, 95% CI = (−0.57, −0.09); moderate, SMD = −0.64, 95% CI = (−0.85, −0.42); high, SMD = −0.25, 95% CI = (−0.44, −0.05)], with moderate-intensity exercise producing the best effect (*p* < 0.05). Only light- and moderate-intensity exercise relieved anxiety after the intervention [light, SMD = −0.48, 95% CI = (−0.71, −0.26); moderate, SMD = −0.58, 95% CI = (−0.85, −0.31)]. Only high-intensity exercise worked in alleviating stress [SMD = −1.13, 95% CI = (−2.22, −0.04)]. Both irritability and restlessness could be improved by light- and moderate-intensity exercise [irritability, SMD = −0.74, 95% CI = (−0.98, −0.50); restless, SMD = −0.72, 95% CI = (−0.98, −0.47)], and there were no statistical differences between the subgroups (*p* > 0.05). Moderate- and high-intensity exercise decreased withdrawal syndrome after the intervention [moderate, SMD = −0.30, 95% CI = (−0.55, −0.05); high, SMD = −1.33, 95% CI = (−1.90, −0.76)], with high-intensity exercise producing the best effects (*p* < 0.01).

**Conclusion:** Overall, exercise leads to improvements in withdrawal symptoms in individuals with SUD, but these effects vary significantly between the exercise of different intensities and according to the type of withdrawal symptoms. Moderate-intensity exercise has the greatest benefits in improving depression and anxiety; high-intensity exercise has the greatest benefits in improving withdrawal syndrome.

**Systematic Review Registration:** www.crd.york.ac.uk/PROSPERO/, identifier, CRD42022343791

## 1 Introduction

Substance use disorder (SUD) is a chronic, relapsing, and serious brain disease characterized by compulsive drug use despite harmful consequences and repeated episodes of intoxication and withdrawal (Navarrete et al., 2021). In the fifth edition of the Diagnostic and Statistical Manual of Mental Disorders (DSM-V), SUD combines categories of substance abuse and substance dependence into a single disorder that can be diagnosed by two or more out of the 11 listed symptoms (Hasin et al., 2013). There are three levels of SUD: mild, moderate, and severe. According to the DSM-V, drug addiction is synonymous with severe SUD, in which affected individuals have poor ability to remain abstinent, despite their strong willingness to discontinue drug use (Hasin et al., 2013). One of the important reasons for this high failure rate of abstinence is the occurrence of severe withdrawal symptoms in people with SUD; these usually present in the forms of craving, depression, anxiety, stress, irritability, restlessness, and withdrawal syndrome. Craving, depression, and anxiety have received the most attention in previous studies, as they are the most common withdrawal symptoms occurring in almost every period of addiction withdrawal. Craving, an intense subjective experience of wanting to use a drug, has been found to be an important indicator in clinical diagnosis and a predictor of treatment outcomes (Venniro et al., 2021). RCTs have demonstrated that exercise interventions can reduce cravings for alcohol (Ussher et al., 2004), nicotine (Prapavessis et al., 2014), and cannabis (Buchowski et al., 2011). Meanwhile, studies in animals have shown that voluntary wheel-running decreases cue-induced cocaine-seeking behavior (Thanos et al., 2013). It should be noted that the exercise-induced anti-craving effects always seem to be transient (Venniro et al., 2021).

Up to 80% of people with SUD have comorbid depression. There is evidence that addressing comorbid depression among people with SUD can adversely affect their long-term prognosis (Tirado Muñoz et al., 2018). Comorbidity of anxiety and SUD is another common phenomenon. One meta-analysis concluded that people with SUD are at a 2.1 times greater risk of having anxiety compared to those without SUD (Lai et al., 2015). Regarding stress, this can exacerbate craving, leading to relapse. In addition, laboratory rats show heightened susceptibility to relapse when exposed to stressors after extended periods of withdrawal and exhibit persistent and heightened expression of stress-induced anxiety (Erb, 2010). Furthermore, a small number of studies have proposed that irritability and restlessness during the withdrawal period play a critical role in relapse (Shahab et al., 2013; Prapavessis et al., 2014). It is noteworthy that withdrawal syndrome is a complex set of symptoms consisting of many negative mood states, including craving, depression, anxiety, and stress, which can be measured using specialized scales. All these symptoms strongly drive the patient to consume the substances of addiction again, and a vicious cycle might be generated. According to a recent global survey, SUD treatment and harm-reduction services were significantly impacted globally during the COVID-19 pandemic (Radfar et al., 2021). This resulted in an increase of relapse rates and elevated risk of drug misuse (United Nations Office on Drugs and Crime, 2022). Therefore, an urgent demand has arisen for effective, safe, and economical treatments for SUD.

There is extensive work in the literature (Centanni et al., 2022; Li et al., 2022; Re et al., 2022) demonstrating that exercise can improve withdrawal symptoms and reduce relapse rates. Therefore, exercise has been considered an excellent adjunct treatment to existing treatment regimens (Wang et al., 2014; He et al., 2021; Salem et al., 2022). However, the detailed mechanisms underlying exercise-induced reductions in relapse through reversal of withdrawal symptoms remain unclear. As mentioned above, there are at least seven common types of withdrawal symptoms; it is also disputable whether exercise can improve all these symptoms to reduce relapse. Several studies have found that exercise may have positive effects on methamphetamine-associated craving and inhibitory control (Wang et al., 2016) and may be associated with better sleep quality in cigarette smokers (Andreassen et al., 2019; Purani et al., 2019). In addition, a meta-analysis (Taylor et al., 2007) revealed that relatively small doses of exercise may be recommended as an aid to managing cigarette cravings and withdrawal symptoms. Contrary to these results, one study has not found any therapeutic effect of exercise on withdrawal symptoms (Ussher et al., 2008). Indeed, most previous studies have focused on whether exercise can improve withdrawal symptoms to reduce relapse. However, the dose–effect relationship between exercise intensity and improvement in withdrawal symptoms has received relatively little attention. The opposite outcomes might just be attributed to some factors, such as addictive substance, exercise modality, exercise parameters (intensity, duration, and frequency), and the measurement time of outcomes. Exercise intensity, duration and frequency are all important in determining the outcomes (exercise induced improvement in withdrawal symptoms), with exercise intensity being the most important one. The latest meta-analysis indicated that exercise is beneficial in improving the physical and mental health of drug addicts (Jia et al., 2022); however, the authors do not discuss the differences in outcome caused by different exercise intensities, which is not conducive to the formulation of precise exercise prescriptions for people with SUD. Preliminary evidence supports the view that moderate-intensity exercise may create more positive effects (Wang et al., 2016), but this result needs to be verified by support from RCTs with larger samples or high evidence-level meta-analyses. Therefore, we performed this meta-analysis to explore the effects of different exercise intensities on withdrawal symptoms in people with SUD.

## 2 Methods

The process for this systematic review and meta-analysis followed the Preferred Reporting Items for Systematic Reviews and Meta-Analyses (PRISMA) guidelines 2020 (Page et al., 2021), and the meta-analysis was registered in the International Prospective Register of Systematic Reviews (PROSPERO, www.crd.york.ac.uk/PROSPERO/) on 13 July 2022 (registration number: CRD42022343791).

### 2.1 Inclusion and exclusion criteria

All articles included in this meta-analysis were required to meet the following criteria: 1) all reported on RCTs among people with SUD; 2) exercise was used as a unique intervention in the experimental group and non-exercise interventions (e.g., health education) were used in the control group; and 3) the outcomes (withdrawal symptoms: craving, depression, anxiety, stress, irritability, restlessness, and withdrawal syndrome) were measured using any standardized neuropsychological scales and reported in the form of means and standard deviations (M ± SD) or other forms that could be transformed to M ± SD. It is necessary to note that four articles (Prapavessis et al., 2014; Lotfalian et al., 2020; Brellenthin et al., 2021; Zhou et al., 2021) presented outcomes in the form of bar or line graphs, meaning that specific data needed to be extracted using Engauge Digitizer 11.3.

Articles that met any of the following criteria were excluded: 1) the participants received psychiatric treatments or suffered from other serious diseases; 2) exercise interventions were combined with other intervention methods (e.g., pharmacological therapy or cognitive-behavioral therapy); 3) animal studies; and 4) conference abstracts, review articles, editorials, and non-English language articles.

### 2.2 Search strategy

All articles published before June 2022 on the effects of exercise on abstinence symptoms among people with SUD were searched in PubMed, EBSCO, Web of Science, and the Cochrane Central Register of Controlled Trials. The following MeSH key words were used in the searches: “Exercise,” “Physical activity,” “Sports”; “Substance-Related Disorders,” “Addiction Medicine,” “Substance Abuse, Intravenous”; “Substance Withdrawal Syndrome,” “Substance Abstinence Syndrome,” “Drug-Seeking Behavior,” “Craving,” “Depression,” “Anxiety,” “Mental Health.” Manual searching of the references of retrieved articles was also performed. Two authors (HL and JC) independently screened the titles, abstracts, or full texts and excluded any irrelevant articles. All studies that qualified for inclusion were uploaded into EndNote 20.3 in order to remove duplicates; subsequently, another two authors (YL and LZ) checked the qualifying studies again. Any disagreements during this process were resolved through discussion.

### 2.3 Data extraction and quality assessment

For each included study, the extracted data included the study name, author information, participants’ characteristics (including sex and age), sample size, abuse substance, exercise type, exercise intensity, exercise frequency, exercise duration, exercise period, the measures used to assess the outcomes, and specific outcome data (M ± SD) for meta-analysis. If an article presented data only in figures (Prapavessis et al., 2014; Lotfalian et al., 2020; Brellenthin et al., 2021; Zhou et al., 2021), we used the Engauge Digitizer 11.3 tool to extract the data from the figures. One author (HL) extracted all the abovementioned information from the 22 included articles, and another author (JC) checked the data extracted, based on the Cochrane Handbook recommendations. Discrepancies in data extraction were discussed among all researchers, and a final decision was then taken.

RoB 2.0 (Sterne et al., 2019) was used to evaluate the quality of eligible studies. The studies were assessed as “low,” “high,” or “some concerns” on each of five categories: 1) randomization process; 2) deviations from the intended interventions; 3) missing outcome data; 4) measurement of the outcome; and 5) selection of the reported result (Cumpston et al., 2019). Two reviewers (HL and WS) used the Cochrane RoB 2.0 to assess RCTs for independent judgements of the quality, and discrepancies were resolved through discussion with a third reviewer (YL) to reach a consensus.

### 2.4 Data synthesis and statistical analyses

The analysis was performed using RevMan 5.3. The included studies reported various measures of withdrawal symptoms, which created the possibility of potential heterogeneity. Thus, we employed SMD with 95% confidence intervals (CIs) to represent the effect size values of all outcomes (which were scale scores, a form of continuous variable). The chi-square test and I^2^ statistic were used to evaluate the heterogeneity of studies. In cases where *p* > 0.05 and I^2^ < 45%, a fixed effects model was used to calculate pooled effect sizes; otherwise, a random effects model was used. For the subgroup analysis, all included studies were divided into three subgroups according to exercise intensity (Norton et al., 2010): low-intensity, moderate-intensity, and high-intensity. A sensitivity analysis was conducted to determine the stability and reliability of the results of this meta-analysis by altering the statistical model and deleting high-heterogeneity studies. In this type of analysis, the absence of significant changes indicates that the meta-analysis is robust, whereas the presence of such changes indicates that it is not robust.

## 3 Results

### 3.1 The process of study selection

**FIGURE 1:**
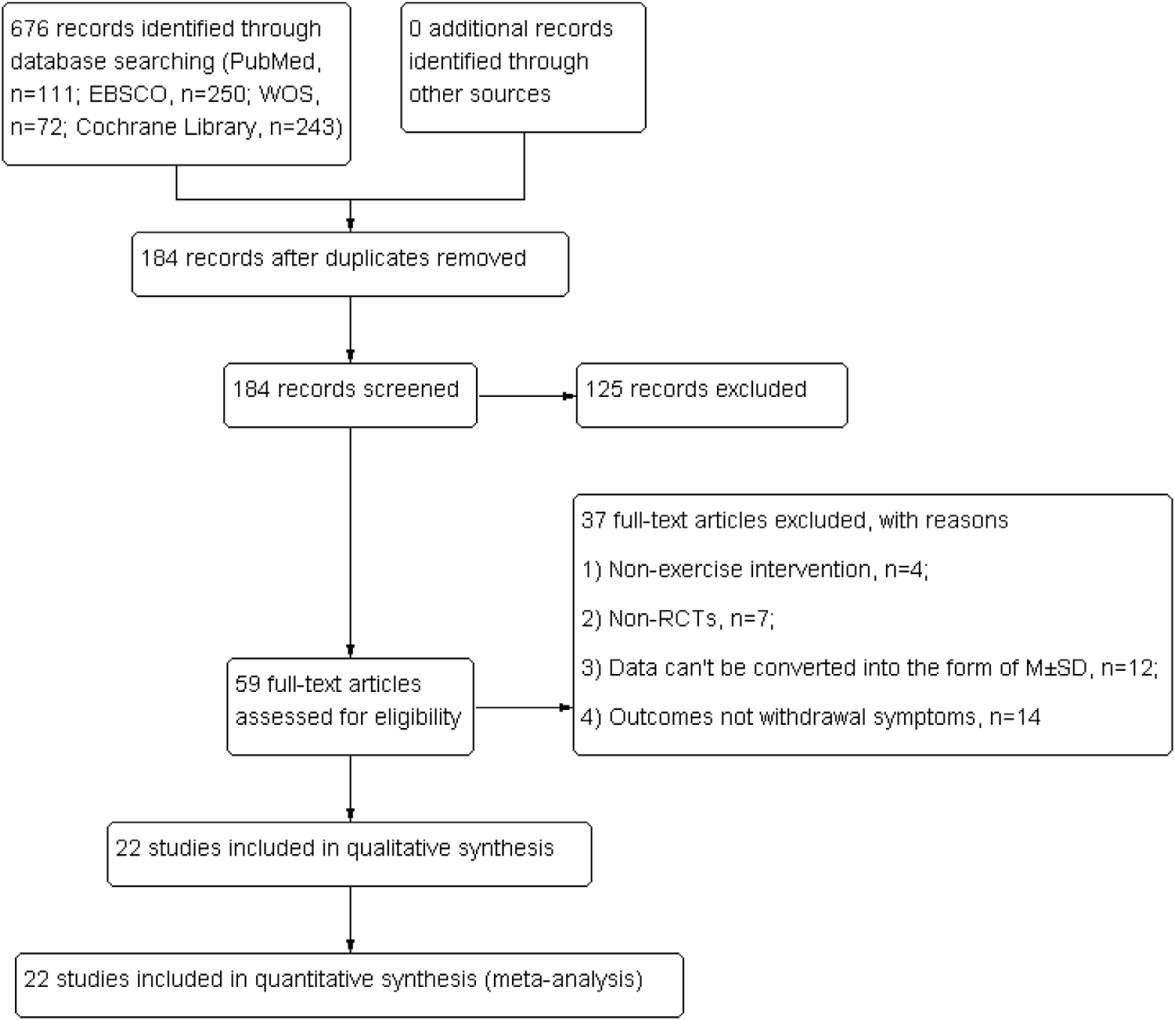
Flow diagram of the process of article selection.

The literature search, identification, and selection process is shown in Figure 1. A total of 676 potentially relevant articles were initially identified in four electronic databases (PubMed, *n* = 111; EBSCO, *n* = 250; WOS, *n* = 72; Cochrane Library, *n* = 243). In total, 184 articles were retrieved after deletion of duplicates. Subsequently, 125 irrelevant articles were removed through screening of the title and abstract. The remaining 59 articles were assessed by reading the full text, and 37 articles did not match the inclusion criteria. Hence, 22 eligible articles were included in this systematic review and meta-analysis.

### 3.2 Characteristics of included studies

The basic characteristics of the 22 RCTs included in the analysis are summarized in Table 1. In total, 15 articles (Ussher et al., 2006; Daniel et al., 2007; Li et al., 2013; Shahab et al., 2013; Abrantes et al., 2014; Prapavessis et al., 2014; Taylor et al., 2014; Rawson et al., 2015; Oh and Kim, 2016; Allen et al., 2018; Lotfalian et al., 2020; Zhang and Zhu, 2020; Hallgren et al., 2021b; Brellenthin et al., 2021; Smits et al., 2021) reported more than one outcome, while 7 articles (Elibero et al., 2011; Tritter et al., 2015; Schneider et al., 2016; Patten et al., 2017; Hallgren et al., 2021a; McCartney et al., 2021; Zhou et al., 2021) explored the effects of an exercise intervention on only one type of withdrawal symptom. All 22 eligible articles were published between 2006 and 2021. A total of 1,537 participants were included, and the sample size of each individual study ranged from 20 to 135. Most of the studies recruited participants of both sexes, but six studies were limited to female or male participants; two studies were unclear on this (Oh and Kim, 2016; Schneider et al., 2016). All participants were above 16 years old. The abuse substances involved in the included studies included nicotine (59.09%), alcohol, cocaine, amphetamine, heroin, and cannabis, and there was one study (Prapavessis et al., 2014) in which the participants were pregnant.

**TABLE 1:** Basic characteristics of included articles

Study number	Source (country)	Abuse substances	Participants (E/C)	Sex	Age ①	Outcome measures ②	Exercise intervention				
							Type	Intervention period	Duration (min/d)	Frequency (d/w)	Intensity ③
1	Ussher et al. (2006) (United Kingdom)	Nicotine	20/20	Both (40% women)	30.50 ± 7.98	MPSS	Isometric exercise	—	5	—	Light
2	Daniel et al. (2007) (United Kingdom)	Nicotine	45/45	Both (51% women)	16–65	MPSS	Bicycling	—	10	—	Moderate (40%-60% HRR)
3	Elibero et al. (2011) (US)	Nicotine	30/30	Only women	41.5 ± 12.3	ADACL	Treadmill running	6 weeks	50	3	Moderate (3-6METs)
4	Li et al. (2013) (CHN)	Heroin	17/16	Only women	30.7 ± 6.3	HRSD, RSHWS	Tai chi	6 months	60	3	Light
5	Shahab et al. (2013) (United Kingdom)	Nicotine	48/48	Both (45.8% women)	33.9 ± 10.4	MPSS	Yogic-style breathing	—	10	—	Light
6	Abrantes et al. (2014) (US)	Nicotine	30/31	Both (65.6% women)	47.3	CES-D, PANAS, MWS	Treadmills	12 weeks	50	2-4	Moderate (55%-69% HRmax)
7	Taylor et al. (2014) (United Kingdom)	Nicotine	49/50	Both	>18	mCEQ, PSS, MPSS	Walking	16 weeks	15	?	Moderate
8	Prapavessisa et al. (2014) (United Kingdom)	Nicotine	14/16	Only women	25.7 ± 5.5	SPPS, MPSS	Treadmill walking	—	20	—	Light (25%-55% HRR)
9	Rawson et al. (2015) (US)	Methamphetamine	69/66	Both (29.6% women)	31.7 ± 6.9	BDI, BAI	Aerobic and resistance	8 weeks	60	3	Moderate (60%-80% HRmax)
10	Tritter et al. (2015) (United Kingdom)	Nicotine	15/15	Both (66.7% women)	38.64 ± 8.25	IHDS	Treadmill	—	15	-	Moderate (45%-68% HRR)
					41.73 ± 12.10						
11	Oh and Kim. (2016) (KR)	Alcohol	19/19	?	45.4 ± 7.8	BDI-II, STAXI-K	Tai chi	8 weeks	30	3	Moderate (RPE11-13)
					48.1 ± 11.5						
12	Schneider et al. (2016) (GER)	Alcohol	34/30	?	18–75	ACQ	Walking	3 weeks	60	3	Moderate
13	Patten et al. (2017) (US)	Nicotine	15/15	Only women	38.0 ± 11.0	PHQ-9	Aerobic exercise	12 weeks	30	3	High (RPE>14)
					37.0 ± 10.0						
14	Allen et al. (2019) (US)	Nicotine	12/11	Both (63% women)	30.3 ± 1	MNWS, QSU, CESD	Bicycling	12 weeks	20	3	High (>90% HRR)
						PNAS, PSS					
		Nicotine	9/11	Both (63% women)	30.3 ± 2	MNWS, QSU, CESD	Jogging	12 weeks	30	3	Moderate
						PANAS, PSS					
15	Zhang and Zhu. (2020) (CHN)	Amphetamine	38/38	Only men	40	BDI, S-AI, T-AI, DSQ	Tai chi	6 months	20	4	Light (48%-55% HRmax)
16	Lotfalian et al. (2020) (US)	Nicotine	20/20	Both (50% women)	40.38 ± 12.79	URS, PANAS	Yogic breathing	—	20	—	Light
17	Smits et al. (2021) (US)	Nicotine	77/73	Both (67.3% women)	38.7 ± 10.3	ASI-3, PROMIS	Aerobic exercise	6 months	25	3	High (60%–85% HRR)
					38.4 ± 10.6						
18	Hallgren et al. (2021b) (SWE)	Alcohol	98/98	Both (71.4% women)	53.7 ± 11.8	DAQ, POMS, STAI-Y1	Bicycling	—	12	—	High (RPE14-16)
19	Brellenthin et al. (2021) (US)	Polydrug use	11/10	Both (42.9% women)	35.1 ± 10.2	PHQ-9, PSS, GAD-7	Treadmill	6 weeks	30	3	Moderate (70%-75% HRmax)
					35.0 ± 7.1						
20	Zhou et al. (2021) (CHN)	Methamphetamine	20/20	Only women	18–45	VAS	Dance	—	30	—	Moderate (65%–75% HRmax)
21	Hallgren et al. (2021a) (SWE)	Alcohol	117/117	Both (68.4% women)	52.7 ± 12.3	DAQ	Bicycling	—	12	—	High (RPE14-16)
22	McCartney et al. (2021) (AUS)	Cannabis	19/12	Both (41.9% women)	32.9	DASS	Cycling	1 week	35	6	Moderate (60% VO2max)

①Mean ± SD; Min–Max; Mean; >Min.

②MPSS, the Mood and Physical Symptoms Scale; ADACL, the Activation–Deactivation Adjective Checklist; HRSD, the Hamilton Rating Scale for Depression; RSHWS, the Rating Scale of Heroin Withdrawal Symptoms; CES-D, the Center for Epidemiological Studies Depression Scale; PANAS, the Positive Affect and Negative Affect Scale; MWS, the Minnesota Withdrawal Scale; mCEQ, the Satisfaction with Smoking Scale; PSS, the Perceived Stress Scale; SPS, the Seven-Point Scale; BDI, the Beck Depression Inventory; BAI, the Beck Anxiety Inventory; IHDS, the statement “I have a desire to smoke”; BDI-II, a Korean version of the Beck Depression Inventory; STAXI-K, the State-Trait Anger Expression Inventory — Korean version; ACQ, Alcohol Craving Questionnaire; PHQ-9, Patient Health Questionnaire; MNWS, Minnesota Nicotine Withdrawal Scale; QSU, Questionnaire of Smoking Urges—Brief; CESD, Center for Epidemiological Study—Depression; S-AI, state anxiety; T-AI, trait anxiety; DSQ, psychological craving; URS, Urge Rating Scale; ASI-3, Anxiety Sensitivity Index-3; PROMIS, Patient-Reported Outcomes Measurement Information System; DAQ, the Desire for Alcohol Questionnaire; POMS-Brief, Profile of Mood States questionnaire; STAI-Y1, the State–Trait Anxiety Inventory; GAD-7, the 7-item Generalized Anxiety Disorder assessment; VAS, Visual Analog Scale; DASS, Depression, Anxiety and Stress Scale.

③Norton K, Norton L, Sadgrove D. (2010). Position statement on physical activity and exercise intensity terminology. J Sci Med Sport. 13 (5), 496–502. doi:10.1016/j.jsams. 2009.09.008.

The types of exercise intervention used in the eligible articles were various, including dance, bicycling, walking, and tai chi. The durations of the exercise interventions ranged from 1 week to 6 months. According to Norton’s research (Norton et al., 2010), exercise intensity was divided into low, moderate, and high. The exercise interventions in almost all the studies were carried out under the supervision of exercise specialist. After the exercise intervention, scales were used as a unique method to measure the participants’ withdrawal symptoms. The participants in the control group were mainly treated as usual or received health education in the form of watching a relevant video, and subsequent comparisons were conducted based on the participants who had been assigned to the exercise intervention vs. those who had not.

### 3.3 Risk of bias and quality assessment

**FIGURE 2:**
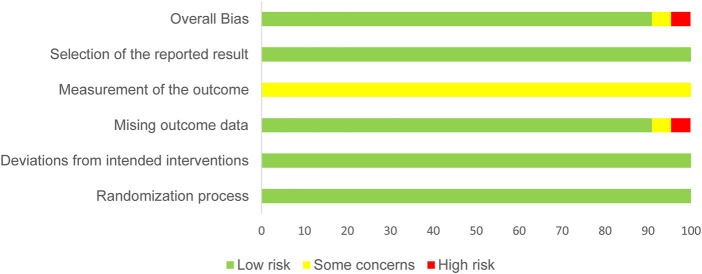
Summary of the assessment of methodological quality using RoB 2.0.

Because all the included studies were RCTs, we selected the RoB 2.0 as a tool to evaluate their methodological quality. The results of this assessment of methodological quality are shown in Figure 2 (the specific evaluation of each study on every domain is shown in Supplementary Figure S1). As shown in the table, although there was no difference between the results of each separate study presented in one article, we also assessed these individually. The aim of the included studies was to determine whether exercise interventions involving exercise of different intensity levels would relieve withdrawal symptoms among people with SUD, so it was not possible for the authors to implement blinding methods (performance bias). Moreover, the outcomes were assessed using scales completed by every participant before and after the intervention, so none of the included trials demonstrated adequate blinding of outcomes assessment (detection bias). According to the Cochrane Collaboration’s guidelines for assessing risk of bias in the Cochrane Handbook for Systematic Reviews of Interventions, the two aforementioned scenarios should be assessed as “low risk.” Discrepancies in the assessment of studies quality were arbitrated by discussion among all authors (HL, WS, JC, LZ, and YL). If one or more domains were rated as “high risk of bias” for a particular study, it was regarded as a low-quality study; if there were one or more domains rated as “unclear risk of bias,” it was regarded as a moderate-quality study; and if all domains were rated as “low risk of bias,” it was regarded as a high-quality study.

### 3.4 Effects of exercise on craving

**FIGURE 3:**
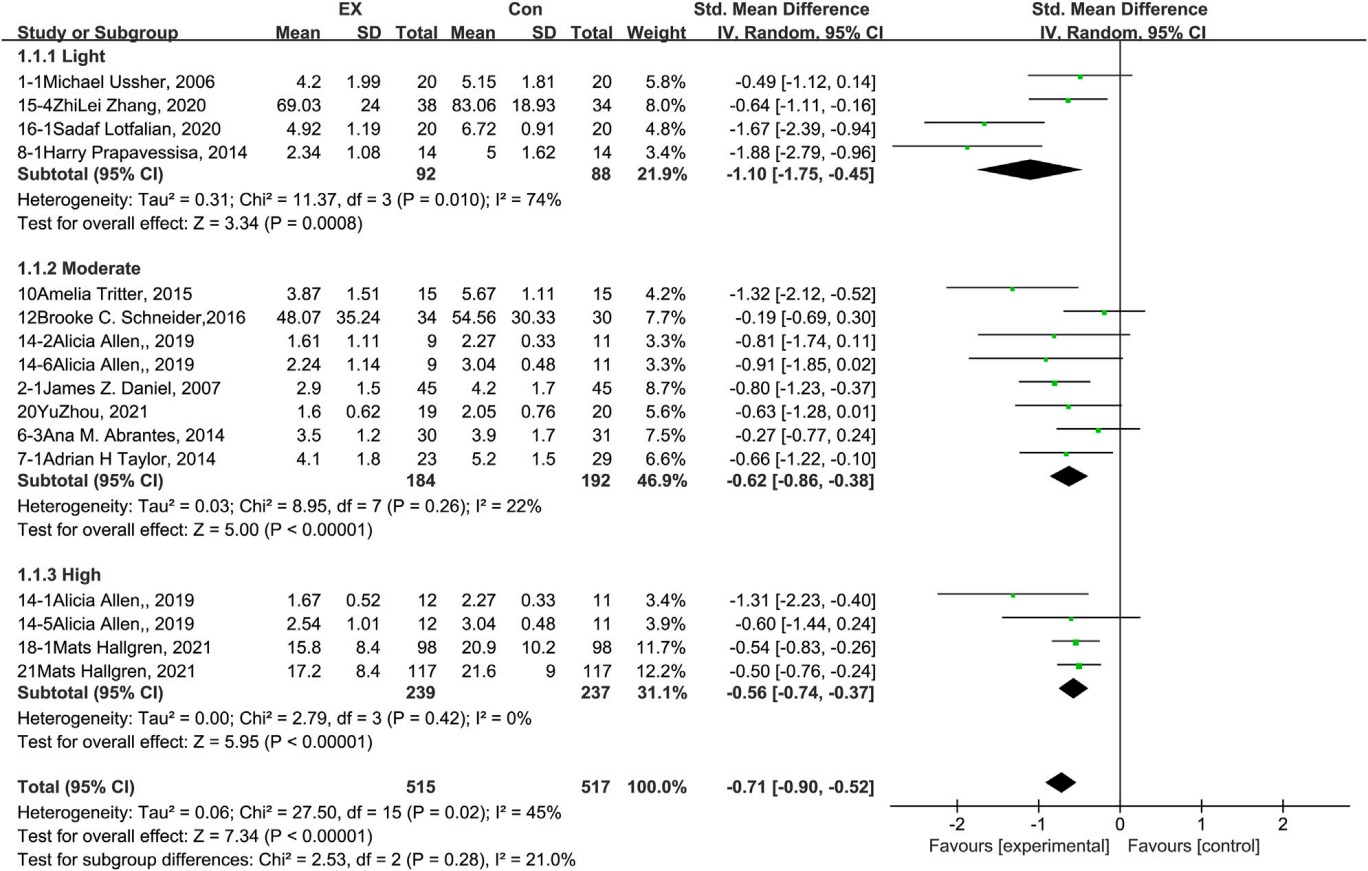
Meta-analysis results on the effects of light-, moderate-, and high-intensity exercise interventions on cravings in people with SUD. Pooled effect sizes were calculated using the random effects model. EX, exercise group; Con, control group; SD, standard deviation; Std, standardized; IV, inverse variance; CI, confidence interval.

The pooled results for effects of exercise on craving are shown in Figure 3. The result of an assessment of publication bias is shown in Supplementary Figure S2. Meta-analysis of 16 trials (four involving exercise of light intensity, eight of moderate intensity, and four of high intensity) demonstrated that exercise interventions reduced cravings among people with SUD during the withdrawal period [*n* = 1,032; SMD = −0.71, 95% CI = (−0.90, −0.52); *p* < 0.05]. These studies exhibited significant statistical heterogeneity (I^2^ = 45%). The results of a sensitivity analysis showed that the heterogeneity was caused by two trials (Prapavessis et al., 2014; Lotfalian et al., 2020) in the light-intensity subgroup, and the pooled result was robust. Subgroup analyses of different exercise intensities showed that there was no significant difference among the three groups in terms of the effects on craving (*p* = 0.28, I^2^ = 21%). Together, these results suggest that exercise induces decreases in craving during the withdrawal period among people with SUD, and the extent of this decrease does not differ among exercise interventions of light, moderate, and high intensity.

### 3.5 Effects of exercise on depression

**FIGURE 4:**
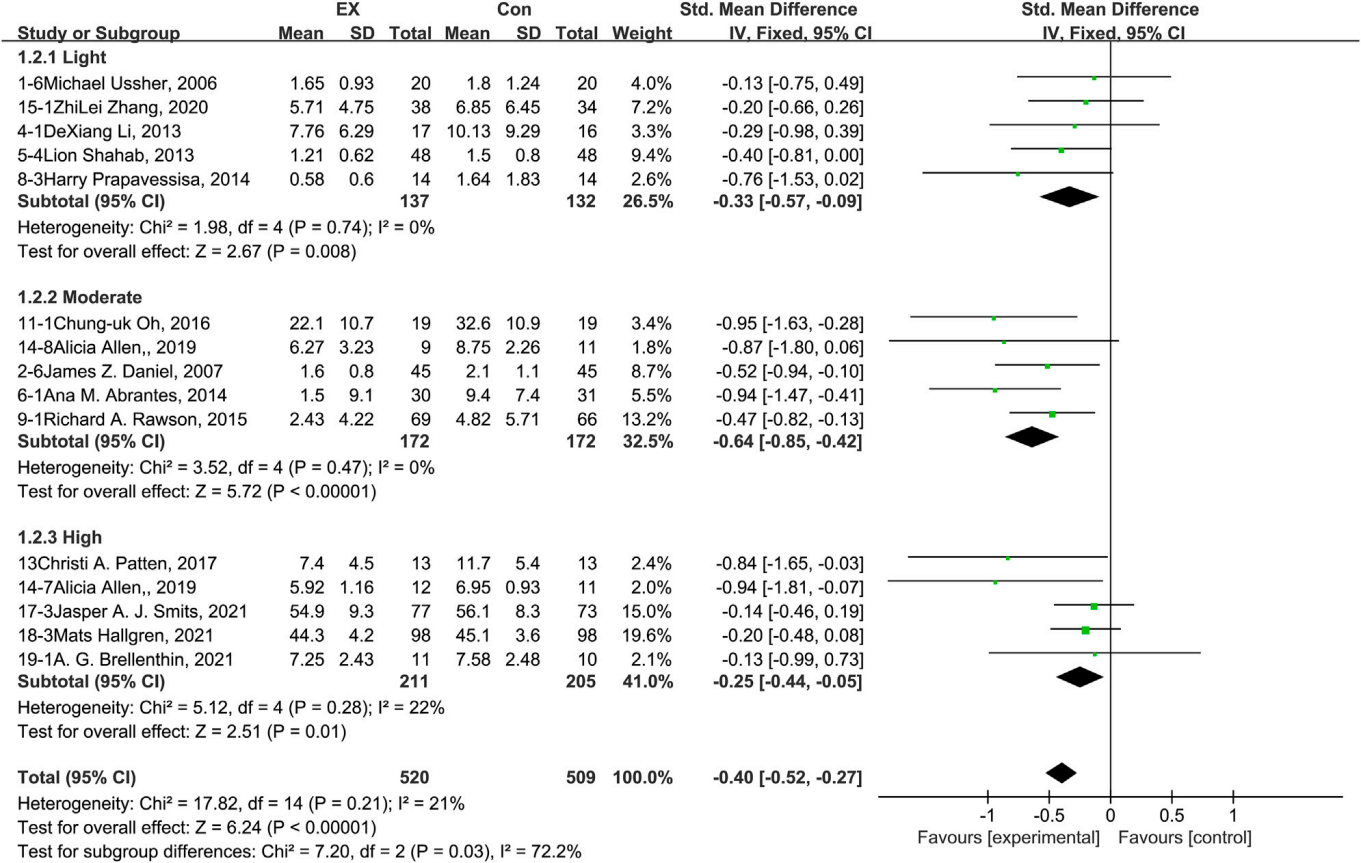
Meta-analysis results on the effects of light-, moderate-, and high-intensity exercise interventions on depression in people with SUD. Pooled effect sizes were calculated using the fixed effects model. EX, exercise group; Con, control group; SD, standard deviation; Std, standardized; IV, inverse variance; CI, confidence interval.

The pooled results for effects of exercise on depression are shown in Figure 4. The result of an assessment of publication bias is presented in Supplementary Figure S3. Meta-analysis of 15 trials (five in each intensity subgroup) demonstrated that exercise relieved depression during the withdrawal period among people with SUD [*n* = 1,029; SMD = −0.40, 95% CI=(−0.52, −0.27); *p* = 0.21, I^2^ = 21%]. Subgroup analyses of different intensities showed that there was a significant difference among the three subgroups in terms of depression outcome (*p* = 0.03, I^2^ = 72.2%). Moderate-intensity exercise produced the best effect [SMD = −0.64, 95% CI=(−0.85, −0.42)]. The sensitivity analysis suggested that this pooled result was robust, given non-subversive results upon changing the effect model or deleting high-heterogeneity studies one at a time. Together, these results suggest that exercise induces remission of depression during the withdrawal period among people with SUD, and the best results can be obtained with a moderate-intensity exercise intervention.

### 3.6 Effects of exercise on anxiety

**FIGURE 5:**
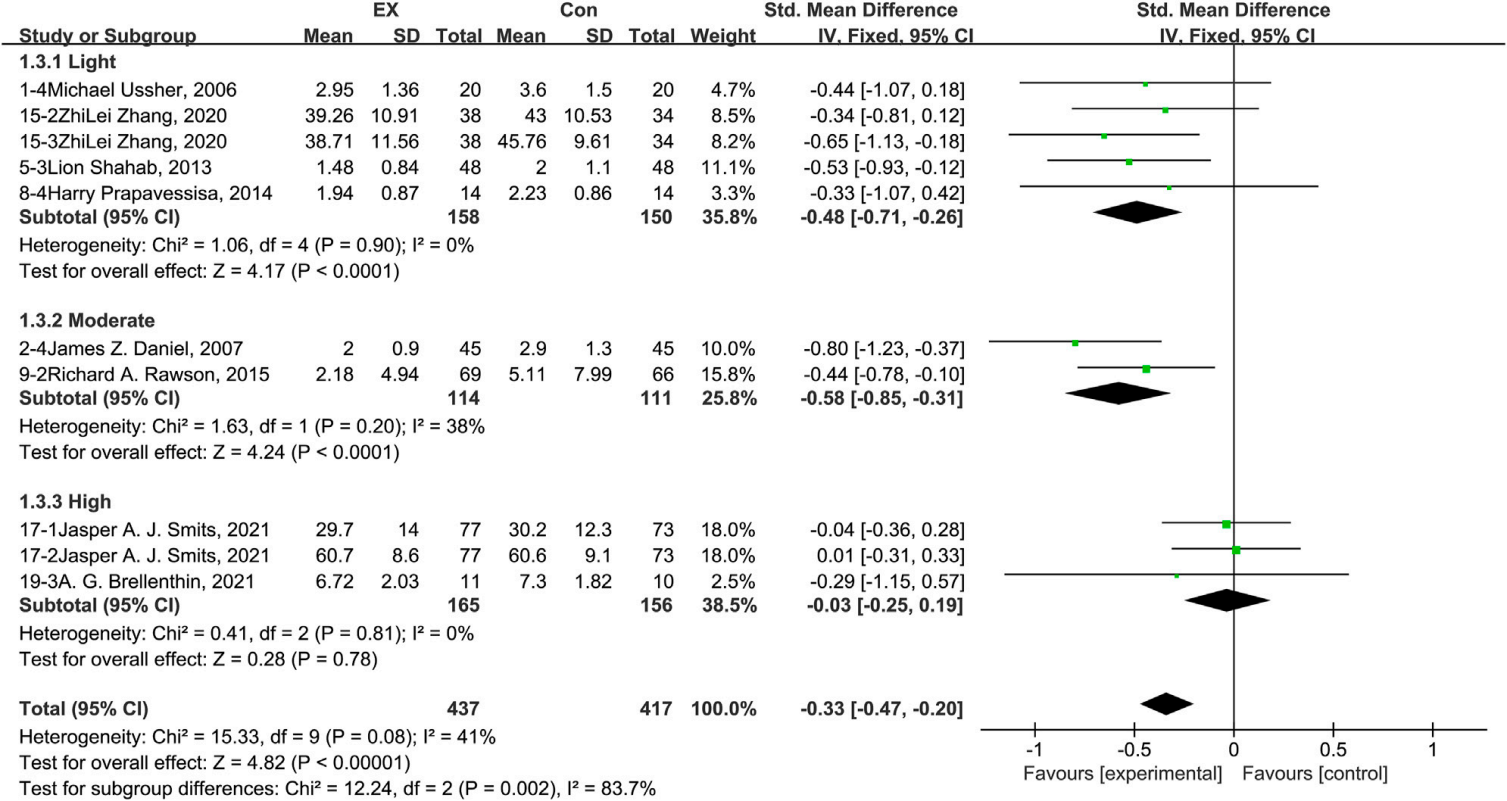
Meta-analysis results on the effects of light-, moderate-, and high-intensity exercise interventions on anxiety in people with SUD. Pooled effect sizes were calculated using the fixed effects model. EX, exercise group; Con, control group; SD, standard deviation; Std, standardized; IV, inverse variance; CI, confidence interval.

The pooled results for effects of exercise on anxiety are shown in Figure 5. The result of an assessment of publication bias is shown in Supplementary Figure S4. Meta-analysis of 10 trials (five in the light-intensity category, two moderate-intensity, and three high-intensity) demonstrated that exercise relieved anxiety during the withdrawal period among people with SUD [*n* = 854; SMD = −0.33, 95% CI=(−0.47, −0.20); *p* = 0.08, I^2^ = 41%]. Subgroup analyses of different intensities showed that there was a significant difference among the three subgroups (*p* = 0.002, I^2^ = 83.7%). Moderate-intensity exercise produced the best effect [SMD = −0.58, 95% CI=(−0.85, −0.31)], while high-intensity exercise was associated with no effect, with high homogeneity [*n* = 321; SMD = −0.03, 95% CI=(−0.25, 0.19); *p* = 0.81, I^2^ = 0%]. The sensitivity analysis suggested that this pooled result was robust, given non-subversive results upon changing the effect model or deleting high-heterogeneity studies one at a time. Together, these results suggest that exercise induces remission of anxiety during the withdrawal period among people with SUD, and the best results can be obtained with a moderate-intensity exercise intervention.

### 3.7 Effects of exercise on stress

**FIGURE 6:**
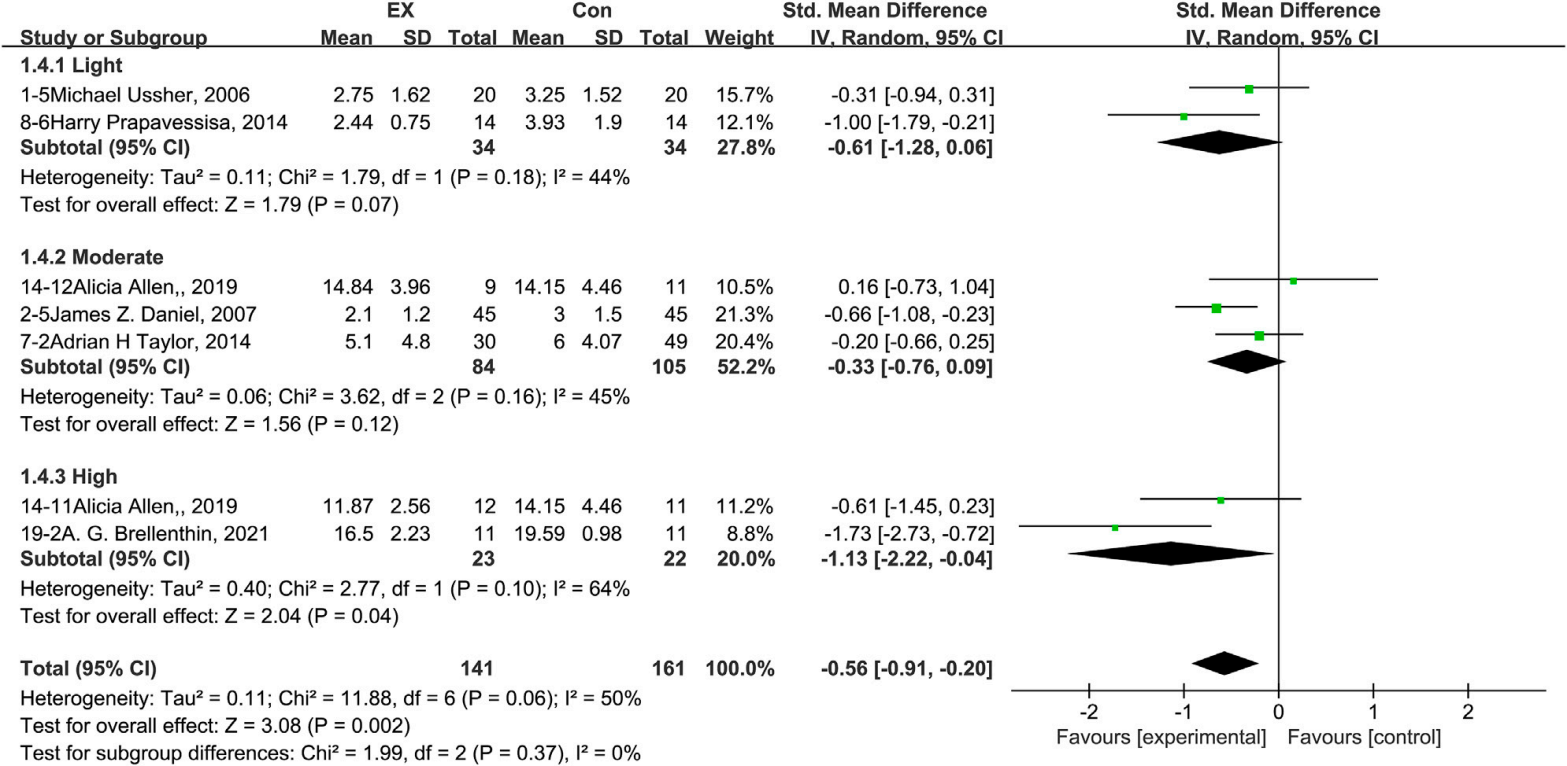
Meta-analysis results on the effects of light-, moderate-, and high-intensity exercise interventions on stress in people with SUD. Pooled effect sizes were calculated using the random effects model. EX, exercise group; Con, control group; SD, standard deviation; Std, standardized; IV, inverse variance; CI, confidence interval.

The pooled results for effects of exercise on stress are shown in Figure 6. The result of an assessment of publication bias is shown in Supplementary Figure S5. Meta-analysis of seven trials (two in the light-intensity category, three moderate-intensity, and two high-intensity) demonstrated that exercise relieved stress during the withdrawal period among people with SUD [*n* = 302; SMD = −0.56, 95% CI=(−0.91, −0.20); *p* = 0.06]; there was statistically significant heterogeneity among the studies (I^2^ = 50%). However, subgroup analysis of different intensities showed that there was no significant difference among the three subgroups (*p* = 0.37, I^2^ = 0%). Together, these results suggest that exercise induces relief from stress during the withdrawal period among people with SUD, and the extent of this relief does not differ among light-, moderate-, and high-intensity exercise interventions.

### 3.8 Effects of exercise on irritability

**FIGURE 7:**
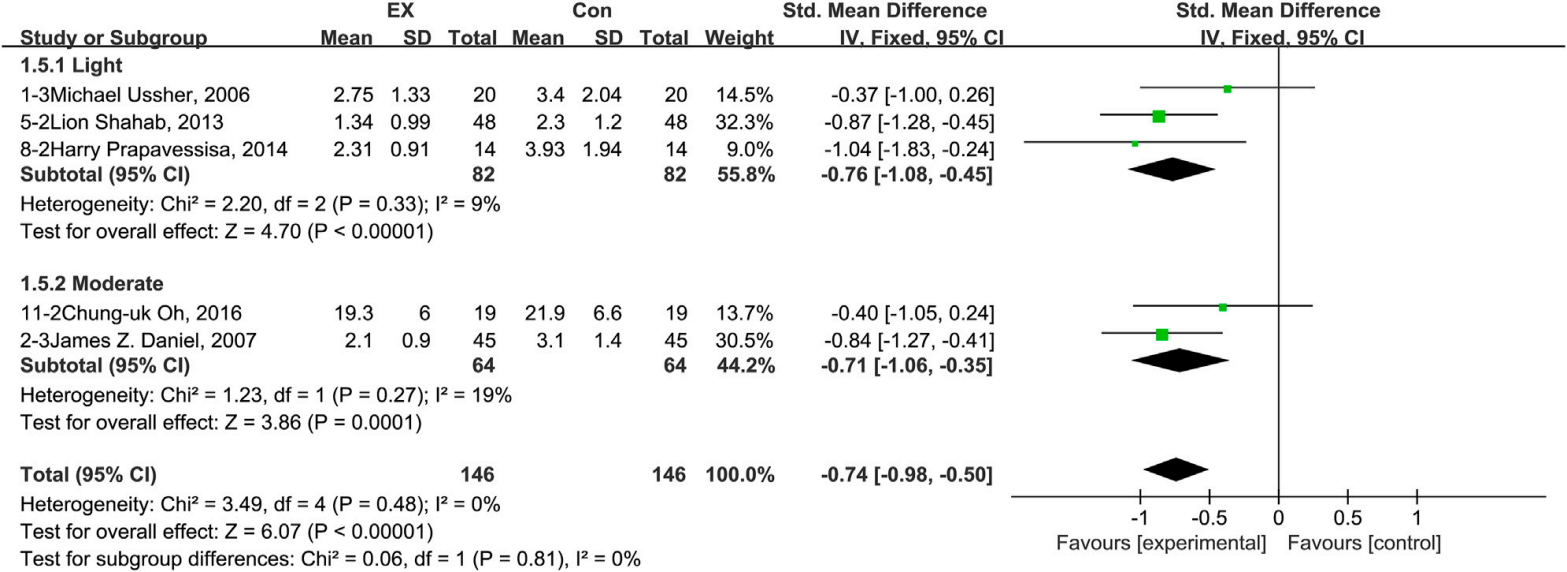
Meta-analysis results on the effects of light- and moderate- intensity exercise interventions on irritability in people with SUD. Pooled effect sizes were calculated using the fixed effects model. EX, exercise group; Con, control group; SD, standard deviation; Std, standardized; IV, inverse variance; CI, confidence interval.

The pooled results for effects of exercise on irritability are shown in Figure 7. The result of an assessment of publication bias is shown in Supplementary Figure S6. Meta-analysis of five trials (three in the light-intensity category and two moderate-intensity) demonstrated that exercise alleviated irritability during the withdrawal period among people with SUD [*n* = 292; SMD = −0.74, 95% CI=(−0.98, −0.50); *p* = 0.48, I^2^ = 0%]. Notably, we did not find any study using high-intensity exercise as intervention to explore the effects of exercise on irritability. Hence, subgroup analyses of different intensities examined only trials involving light- and moderate-intensity exercise. The results showed that there was no significant difference between these two subgroups (*p* = 0.81, I^2^ = 0%). Together, these results suggest that exercise induces alleviation of irritability during the withdrawal period among people with SUD, and the extent of this reversion does not differ between exercise of light and moderate intensity.

### 3.9 Effects of exercise on withdrawal syndrome

**FIGURE 8:**
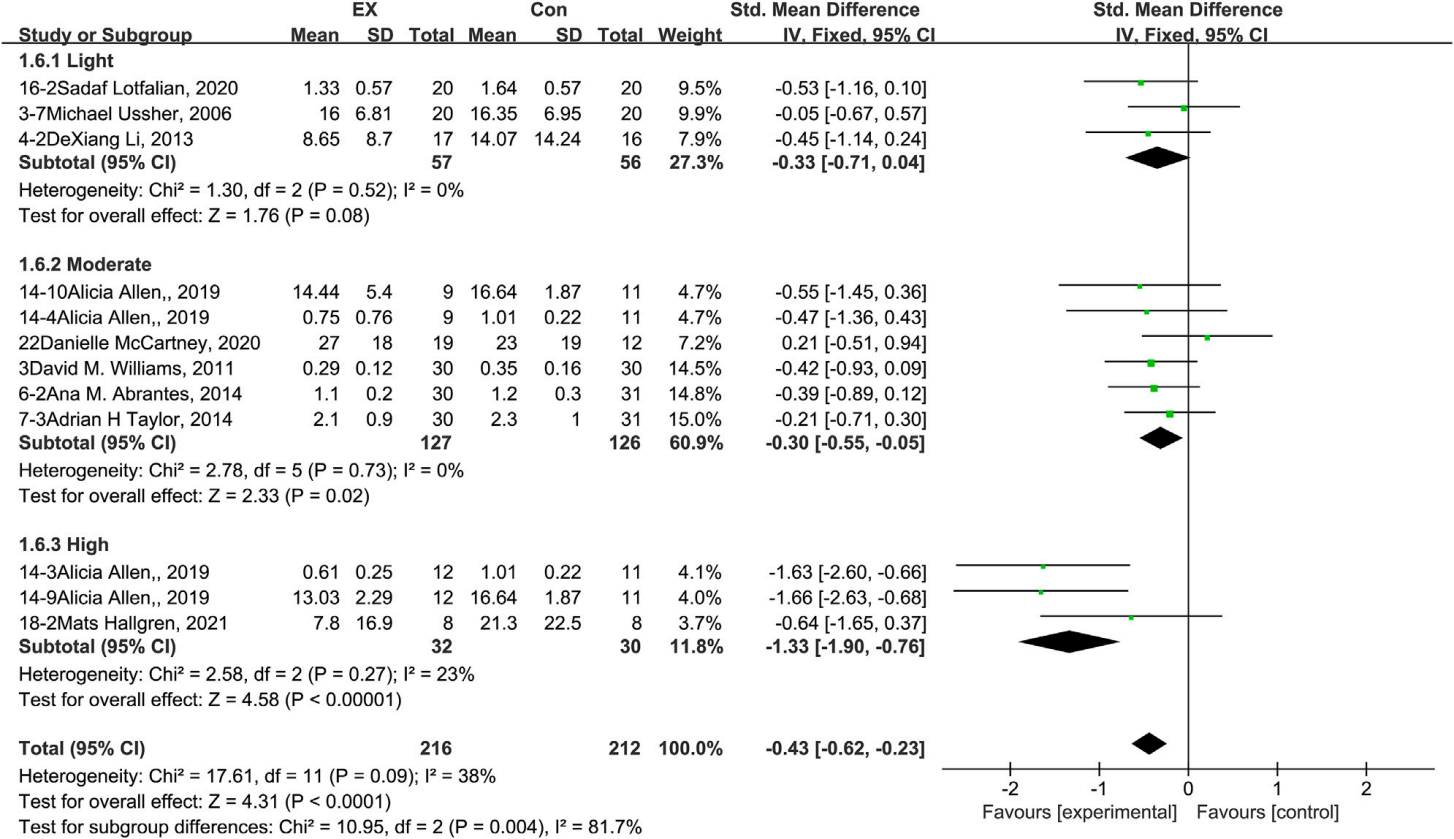
Meta-analysis results on the effects of light-, moderate-, and high-intensity exercise interventions on withdrawal syndrome in people with SUD. Pooled effect sizes were calculated using the fixed effects model. EX, exercise group; Con, control group; SD, standard deviation; Std, standardized; IV, inverse variance; CI, confidence interval.

The pooled results for effects of exercise on withdrawal syndrome are shown in Figure 8. The result of an assessment of publication bias is shown in Supplementary Figure S7. Meta-analysis of 12 trials (three in the light-intensity category, six moderate-intensity, and three high-intensity) demonstrated that exercise relieved withdrawal syndrome during the withdrawal period among people with SUD [*n* = 428; SMD = −0.43, 95% CI = (−0.62, −0.23); *p* = 0.09, I^2^ = 38%]. Subgroup analyses of different intensities showed that there was a significant difference among the three subgroups (*p* = 0.004, I^2^ = 81.7%), with high-intensity exercise producing the largest effect size [SMD = −1.33, 95% CI = (−1.90, −0.76)]. Together, these results suggest that exercise induces relief in withdrawal syndrome during the withdrawal period among people with SUD, and the best results can be obtained with a high-intensity exercise intervention.

### 3.10 Effect of exercise on restlessness

**FIGURE 9:**
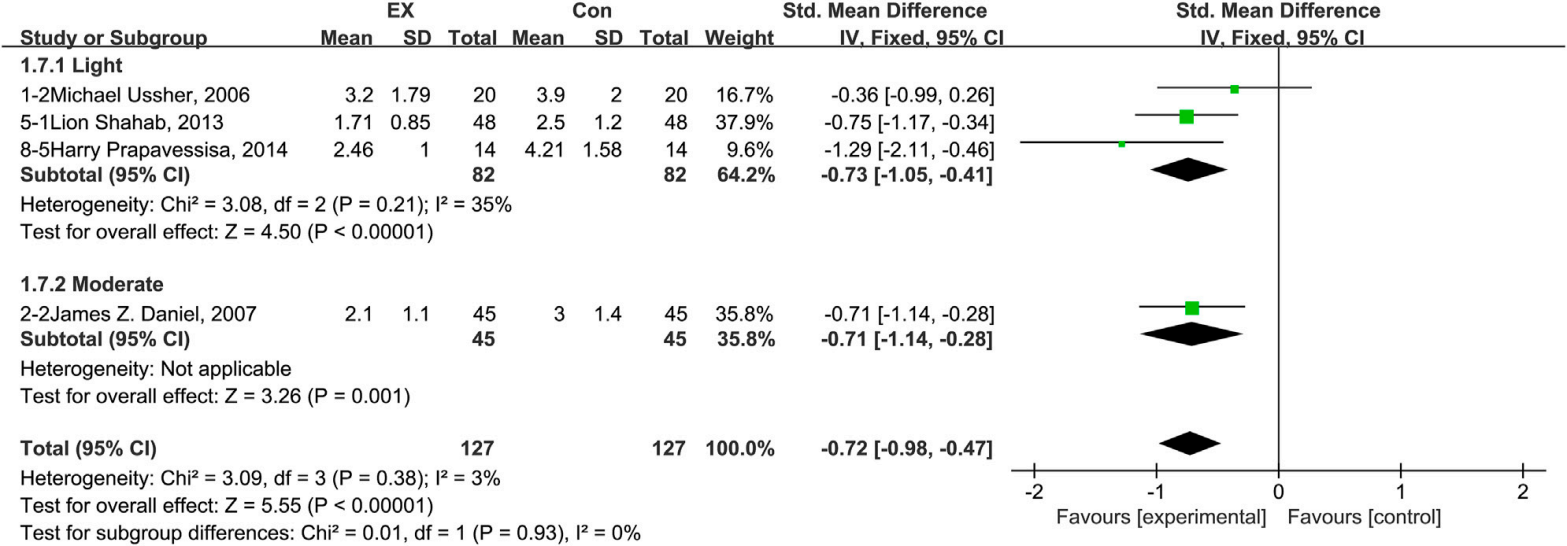
Meta-analysis results on the effects of light- and moderate-intensity exercise interventions on restlessness in people with SUD. Pooled effect sizes were calculated using the fixed effects model. EX, exercise group; Con, control group; SD, standard deviation; Std, standardized; IV, inverse variance; CI, confidence interval.

The availability of publications in the literature on the effects of exercise on restlessness was very sparse. Only four trials (three in the light-intensity category and one moderate-intensity) were included. Therefore, we analyzed only the overall effect size to determine whether exercise can improve restlessness. The pooled results are shown in Figure 9. The result of an assessment of publication bias is shown in Supplementary Figure S8. The results of the meta-analysis showed that exercise improved restlessness during the withdrawal period among people with SUD [*n* = 254; SMD = −0.72, 95% CI = (−0.98, −0.47); *p* = 0.38, I^2^ = 3%].

## 4 Discussion

### 4.1 A brief interpretation of the results

In this meta-analysis, we explored the effects of exercise of different intensities on withdrawal symptoms among people with SUD. The results of our meta-analysis demonstrated that exercise interventions produced improvements in all withdrawal symptoms (craving, depression, anxiety, stress, irritability, withdrawal syndrome, and restlessness) included in this meta-analysis. Furthermore, different exercise intensities were associated with effect of different sizes in the cases of depression, anxiety, and withdrawal syndrome. For depression and anxiety, moderate-intensity exercise interventions produced the best effects. However, for withdrawal syndrome, high-intensity exercise interventions produced the best effects.

During the process of article retrieval, we identified one recent meta-analysis (Jia et al., 2022) that had a similar purpose to the present study. However, there were two limitations to this previous study. First, the type of exercise intervention included in this study was limited to traditional Chinese health-promoting exercises. Second, the intensity of exercise interventions was not considered in this study, although the response to exercise might vary depending on exercise intensity. Our findings replicate those of a previous meta-analysis showing that exercise interventions can induce improvement in withdrawal symptoms. Moreover, we compare studies that followed participants taking part in interventions involving exercise of different intensities, demonstrating that variable effect sizes of exercise for depression, anxiety, and withdrawal syndrome can be distinguished based on exercise intensity (light, moderate, and high).

### 4.2 Possible mechanisms underlying the results

The above findings should be interpreted with caution, because potential biological mechanisms underlying the effects of exercise of different intensity levels on intervention outcomes remain relatively unexplored (Brellenthin et al., 2021).

First, the occurrence of negative emotional states, such as depression and anxiety, during the withdrawal period is attributed to dysfunction of the dopamine reward system. Biological studies have shown that exercise activates the dopamine reward circuits and thus contributes to the reduction of negative emotional states and relapse during the withdrawal period. The effects of exercise on brain functions often do not follow a linear course (Sarbadhikari and Saha, 2006), and higher-intensity exercise is associated with more negative affect during exercise (Bixby et al., 2001). However, limited research has been conducted concerning the effect of exercise intensity on the activation of the dopamine reward system. Recent studies have shown that exercise enhances activation of dopamine neurons in the ventral tegmental area (VTA) by the red nucleus–VTA glutamate pathway (He et al., 2022) and the cerebellum–VTA glutamate pathway (Carta et al., 2019). These might be the neural circuit mechanisms that are responsible for exercise-induced protection against addiction and withdrawal. However, this finding is contrary to that of Jung et al. (2021), who found that exercise increased striatal dopamine levels in normal animals but did not affect the dopaminergic signaling pathway in animals administered methamphetamine. Thus, further research is necessary, focusing on the effects of exercise of different intensity levels on dopamine release in addiction and withdrawal patients or in animal models.

Second, studies have demonstrated that the endocannabinoid (eCB) system is inhibited in SUD patients, which might contribute to various types of withdrawal symptoms. An exercise intervention may be considered to be a non-pharmacological way to augment the eCB system, due to its efficacy in reliably increase the circulation of eCBs in heathy individuals (Hill and Patel, 2013; Morgan et al., 2013; Brellenthin et al., 2017). A previous study has shown that changes in eCB signaling are dependent on exercise intensity (Meyer et al., 2019). Significant changes in circulating eCBs are observed after moderate-intensity exercise; however, the level of circulating eCBs might not be changed after very low- or very high-intensity exercise (Raichlen et al., 2013). A recent meta-analysis showed that moderate-intensity exercise is associated with greater increases in anandamide (AEA) when compared with low-intensity exercise (Desai et al., 2022). Additionally, in a human study, significant increases in serum concentrations of AEA and oleoylethanolamide were observed in women with major depressive disorder after a moderate-intensity exercise intervention (RPE 13.1, HR 126.7 bpm, 4.0 MET) (Meyer et al., 2019). Furthermore, AEA increases with increasing exercise intensity within the range of moderate intensity (Marin Bosch et al., 2020). These results may help to explain why moderate-intensity exercise interventions have the best outcomes in attenuation of depression and anxiety among people with SUD. In contrast, a recent study has demonstrated that the cannabinoid 1 receptor and the cannabinoid 2 receptor are activated after high-intensity swimming exercise above the maximum lactate steady-state intensity (Ludtke et al., 2020). However, the increased level of AEA induced by vigorous exercise is lower than that induced by moderate-intensity exercise. This substantial heterogeneity might depend on exercise intensity, physical fitness, timing of measurements, and/or fasted state (Desai et al., 2022).

It is important to point out that the aforementioned results were not obtained from people or animals with SUD. Thus, further research should be performed on the effects of exercise intensity on the eCB system during the withdrawal period in patients with SUD or in animal models of SUD. In summary, the role of exercise interventions in the treatment of addiction and withdrawal is complex. More studies focusing on the effects of different exercise interventions on neural adaption and disorders during addiction reward and withdrawal would clarify the relevant associations and mechanisms.

### 4.3 Three key factors affecting the outcomes

#### 4.3.1 Different abuse substances

In reality, the effects of an exercise intervention on different withdrawal symptoms may be influenced by several interacting factors, such as the types of addictive substances involved. The substances used in the studies included in our review included nicotine, alcohol, cannabis, heroin, and amphetamine. The addictive mechanisms of these substances are not exactly the same.

For example, the addiction mechanism differs between nicotine and methamphetamine (METH), which were two of the most commonly used substances in these studies. Nicotinic acetylcholine receptors (nAChRs) in the reward circuits of the brain constitute the potential targets of nicotine addiction and withdrawal (Prochaska and Benowitz, 2019; Picciotto and Kenny, 2021). nAChRs have a large number of subunits, of which α4β2 nAChRs mediate many behaviors related to nicotine addiction (Picciotto and Kenny, 2021). Chronic repeated administration of nicotine results in upregulation of nAChRs, which leads to receptor desensitization (Henderson and Lester, 2015). Furthermore, in addition to the presynaptic nAChRs, nicotine also stimulates the postsynaptic nAChRs in the dopamine neurons of the VTA, enhancing the release of dopamine in the nuclear accumbens, which is thought to be responsible for reinforcing dependence of nicotine (Tiwari et al., 2020). In a previous study, it has been shown that, compared with light- and high-intensity exercise interventions, a moderate-intensity exercise intervention has the most positive effect in terms of improving cognitive dysfunction and perturbed expression of nAChRs and downstream signaling molecules in the prefrontal cortex, which are important for depression and anxiety (Zhou et al., 2018). This result might indicate a possible mechanism contributing to the finding in the present meta-analysis, in which moderate-intensity exercise was found to have the best attenuating effect on depression and anxiety in people with SUDs.

Another important mechanism is that addiction to and withdrawal from METH are related to the pathological release of dopamine (DA), serotonin, and norepinephrine. METH inhibits vesicular monoamine transporter-2 (VMAT2) and activates the DA transporter to reverse DA transport, which results in the redistribution of DA from synaptic vesicles to the cytosol (Panenka et al., 2013). A recent study using qRT-PCR and bioinformatics analysis has revealed that METH addiction downregulates GABAAα1 through miR-181a-mediated regulation of endoplasmic reticulum-associated degradation (ERAD) (Wang et al., 2021). A previous study has found that high-intensity exercise increases GABA concentration in the cortex. Furthermore, the increase in the level of GABA is positively correlated with an increase in blood lactate (Coxon et al., 2018). As a cost-effective, flexible, and accessible method, exercise can restore neurochemical balance, thereby stabilizing the blood–brain barrier and correcting alterations in neurogenesis and gliogenesis in METH users (Morais et al., 2018). Recent studies have shown that high-intensity exercise induces more cerebral oxygenation changes in the prefrontal cortex and left dorsolateral prefrontal cortex during exercise and a stronger positive connection between the orbital frontal cortex and left dorsolateral prefrontal cortex in METH addiction patients, as compared with moderate-intensity exercise (Gao et al., 2022). In animal studies, it has been shown that high-intensity exercise enhances neuroplasticity to a greater extent than moderate-intensity exercise (Andrews et al., 2020; Hugues et al., 2022). Taken together, these findings may further explain the result of the present meta-analysis in which a high-intensity exercise intervention might be more helpful for the improvement of withdrawal syndrome in METH addiction patients as compared with light- and moderate-intensity exercise interventions.

Beyond this, compared with light-intensity exercise in methamphetamine dependence, moderate- and high-intensity exercise have been found to exhibit more beneficial effects on craving (Wang et al., 2016), which appears to contradict the first result of the present study. However, this is not the case; this disparity again simply emphasizes the point that the optimal exercise intensity for an intervention may differ for different abuse substances. Therefore, it should be noted that, due to the existence of different types of addictive substance, the specific mechanism by which exercise improves withdrawal symptoms in people with SUD is still not fully understood.

Furthermore, there was only one included study (Brellenthin et al., 2021) in which the participants were polydrug users, despite the fact that substance abuse is rarely limited to a single drug. In fact, polydrug use is the norm among the population with SUD (Spagnolo et al., 2013). The efficacy of pharmacological treatment for polydrug use is not clear, and more original studies are needed (Hazani et al., 2022). Based on the results of the 19th article included in this study, it seems that high-intensity exercise improves withdrawal symptoms in people with polydrug abuse disorders as it does in patients with single-drug abuse disorders. However, there was only one study including polydrug abuse patients; we look forward to further studies, involving more cases, on the effect of exercise interventions in patients with polydrug abuse disorders.

#### 4.3.2 Short- and long-term exercise interventions

Nine articles included in this meta-analysis (Ussher et al., 2006; Daniel et al., 2007; Shahab et al., 2013; Prapavessis et al., 2014; Tritter et al., 2015; Lotfalian et al., 2020; Hallgren et al., 2021a; Hallgren et al., 2021b; Zhou et al., 2021) examined short-term exercise interventions; the remainder (thirteen articles) examined long-term exercise interventions. Chronic exercise interventions specify a target intensity, and the long-term exercise protocol allows participants to gradually reach this target, maintain it for a period time, and then gradually decrease intensity to complete the exercise intervention. Thus, it was considered reasonable to use the target intensity of a long-term exercise intervention for the meta-analysis. Since the present meta-analysis primarily focused on the efficacy of exercise of different intensities in mitigating withdrawal symptoms, studies involving both short- and long-term exercise interventions were included. However, this might result in potential differences in the outcomes. Although a single bout of exercise induces system-wide responses in humans (Contrepois et al., 2020) and induces an antidepressant-like effect (Morikawa et al., 2021), acute exercise-induced improvement in cognitive function is transient. In contrast, long-term exercise interventions regulate neuroplasticity and induce long-term improvement in brain function, which helps to prevent and repair the damage caused by some neurological diseases (Walsh and Tschakovsky, 2018). It has been confirmed that the rapamycin (mTOR) pathway, which is necessary for neuronal activation and axonal myelination, could be regulated by long-term exercise interventions (Chen et al., 2019). Our previous studies have demonstrated that a long-term, moderate-intensity exercise intervention regulates synaptic plasticity to improve neurological functions in models of aging, Alzheimer’s disease, and hypertension (Li et al., 2017a; Li et al., 2017b; Li et al., 2019; Mu et al., 2022). We have also shown that there is exercise frequency dependence in chronic exercise-induced improvement in synaptic plasticity (Li et al., 2017b). Moreover, our previous studies have suggested that a long-term exercise intervention reverses alcohol abuse-induced brain impairments and cognitive dysfunction (Guo et al., 2022). In summary, compared with short-term exercise interventions, long-term exercise interventions might be more effective in terms of the improvement of brain functions. Regrettably, there is no study investigating the differences between short- and long-term exercise in improving withdrawal symptoms. More specific empirical studies are needed on this issue.

#### 4.3.3 Gender of the patient with SUD

Only six of the included articles reported on studies in which the gender of participants was controlled (Elibero et al., 2011; Li et al., 2013; Prapavessis et al., 2014; Patten et al., 2017; Zhou et al., 2021), so performing a subgroup analysis based on gender would not be practical. In one study, no gender differences in abstinence-induced negative affect were found among non-Hispanic African American smokers (Pang et al., 2019). However, important gender differences in drug addiction and withdrawal have been identified in various other studies. One previous study has demonstrated that male and female adolescents receiving treatment for withdrawal exhibit differences in substance use characteristics (Dean et al., 2010). In addition, a nicotine withdrawal study using longitudinal epidemiologic data has suggested that women are more likely than men to experience withdrawal symptoms and relapse. However, the relationship between experience of withdrawal symptoms and reduced likelihood of reducing smoking is stronger in men (Weinberger et al., 2016). Similarly, a previous study has demonstrated that, when compared with men, women exhibit more rapid escalation from casual drug use to addiction, more severe withdrawal symptoms, and a stronger desire to use drugs due to cue-induced craving (Becker, 2016). The involvement of key neural systems (dopamine, *μ* opioid receptors, kappa opioid receptors, and brain-derived neurotrophic factor) in addiction and withdrawal differs greatly between male patients and female patients, which could explain the gender differences in clinical symptoms and treatment efficacy during withdrawal (Becker and Chartoff, 2019). Moreover, a recent study has shown that gender differences also exist in exercise-induced hypertrophy and gains in maximal strength and flexibility (Warneke et al., 2022). Taken together, the possibility of gender differences in exercise-induced improvement in withdrawal symptoms should be a significant concern. More original studies are needed to further verify the results.

### 4.4 Limitations and future prospects

There may be a few limitations to this systematic review and meta-analysis. First, although the heterogeneity between the included studies was within the acceptable range, the use of different periods of exercise intervention and different forms of control group intervention may potentially impact outcomes. In some studies, negative affect may be aggravated under a completely passive control condition. Moreover, some researchers have even criticized the use of health education lectures as a control intervention as being inappropriate and overly contrasting (Ussher et al., 2006). Second, there was a certain amount of overlap between the intensity of exercise that occurred within the three subgroups, although we divided the studies into these subgroups based on both subjective measures (such as Borg’s RPE scales) and objective measures (such as a percentage of maximal heart rate, heart rate reserve, and METs). We comprehensively considered all the methods used for representation of exercise intensity by the studies and then classified each of the interventions as light-intensity (RPE≤10, METs≤3, HRmax≤55%, HRR≤40%, VO_2_max≤40%), moderate-intensity (10< RPE<14, 3< METs<9, 55%< HRmax<90%, 40%< HRR<85%, 40%< VO_2_max<85%), or high-intensity (RPE≥14, METs≥9, HRmax≥90%, HRR≥85%, VO_2_max≥85%) exercise. This approach reduced the statistical power and neglected differences within each intensity subgroup. Third, in the present meta-analysis, we mainly focused on the intensity of the exercise interventions and did not consider the duration, frequency, type, or period of the exercise interventions. Finally, most studies involving a light-intensity intervention that were included in the present meta-analysis used tai chi and yoga. These forms of exercise combine specific physical postures, breathing techniques, stretching, and meditation (Mohammad et al., 2019). Compared with the physiological responses that occur during endurance exercise (bicycling, treadmill exercise, dance, or walking), tai chi and yoga focus more on coordination and cooperation of the body and mind. Moreover, there is a major difference between light-intensity exercise (tai chi, isometric exercises, or yoga) and moderate- or high-intensity exercise, in terms of motor coordination, motor learning, and concentration, that go beyond the exercise intensity itself. Despite this, the present meta-analysis focused only on the intensity of tai chi and yoga, ignoring other advantages exclusive to these forms of exercise.

In the future, related studies should pay close attention to the inclusion of certain types of withdrawal symptoms, certain intensity levels of exercise, and certain abuse substances; additionally, outcomes should be observed over the course of long-term tracking. A promising avenue would be for researchers to carry out highly collaborative multicenter clinical exercise trials to upgrade the level of evidence: in other words, to obtain results that can better guide the practice of exercise-induced detoxification. In addition, the importance of light-intensity exercise should not be ignored, even though the effect of light-intensity exercise is less strong than that of moderate- and high-intensity exercise. For the treatment of SUD in motor-disabled people (older people, disabled people, and pregnant women), light-intensity exercises, such as yoga breathing, isometric exercises, and tai chi, are easy to follow.

## 5 Conclusion

In summary, according to the results of this review, exercise could be identified as an effective way to improve substance withdrawal symptoms. For depression and anxiety, the best improvement effects are obtained with moderate-intensity exercise interventions. However, for withdrawal syndrome, the best improvement effects are obtained with high-intensity exercise interventions. Furthermore, for cravings, stress, irritability, and restlessness, the effects of light-, moderate-, and high-intensity exercise interventions are consistent. On these grounds, the specific, cardinal phenotype of substance withdrawal symptoms must be considered when creating exercise prescriptions for people with SUD. However, in this meta-analysis, stratification of exposure level (exercise intensity) led to a reduction of statistical power and obscuring of potential differences existing within each exposure level. A dose–response meta-analysis will be needed in the future to provide stronger evidence for treatment of SUDs with exercise.

## Data Availability

The original contributions presented in the study are included in the article/Supplementary Materials, further inquiries can be directed to the corresponding author.
