# Supplementary material for: Effects of exercise of different intensities on withdrawal symptoms among people with substance use disorder: a systematic review and meta-analysis
Source: Front Physiol. 2023 May 10;14:1126777. doi: 10.3389/fphys.2023.1126777 (PMC10208401; doi:10.3389/fphys.2023.1126777)
Supplement: Supplementary file 1 [file DataSheet1.docx]

Supplementary Material

Effect of different exercise intensities on withdrawal symptoms among people with substance use disorder: a systematic review and meta-analysis

Hao Li, Wantang Su, Jiajia Cai, Li Zhao, Yan Li*

*** Correspondence:** Yan Li: bsuliyan@bsu.edu.cn

# Supplementary Figures and Tables

## Supplementary Figures


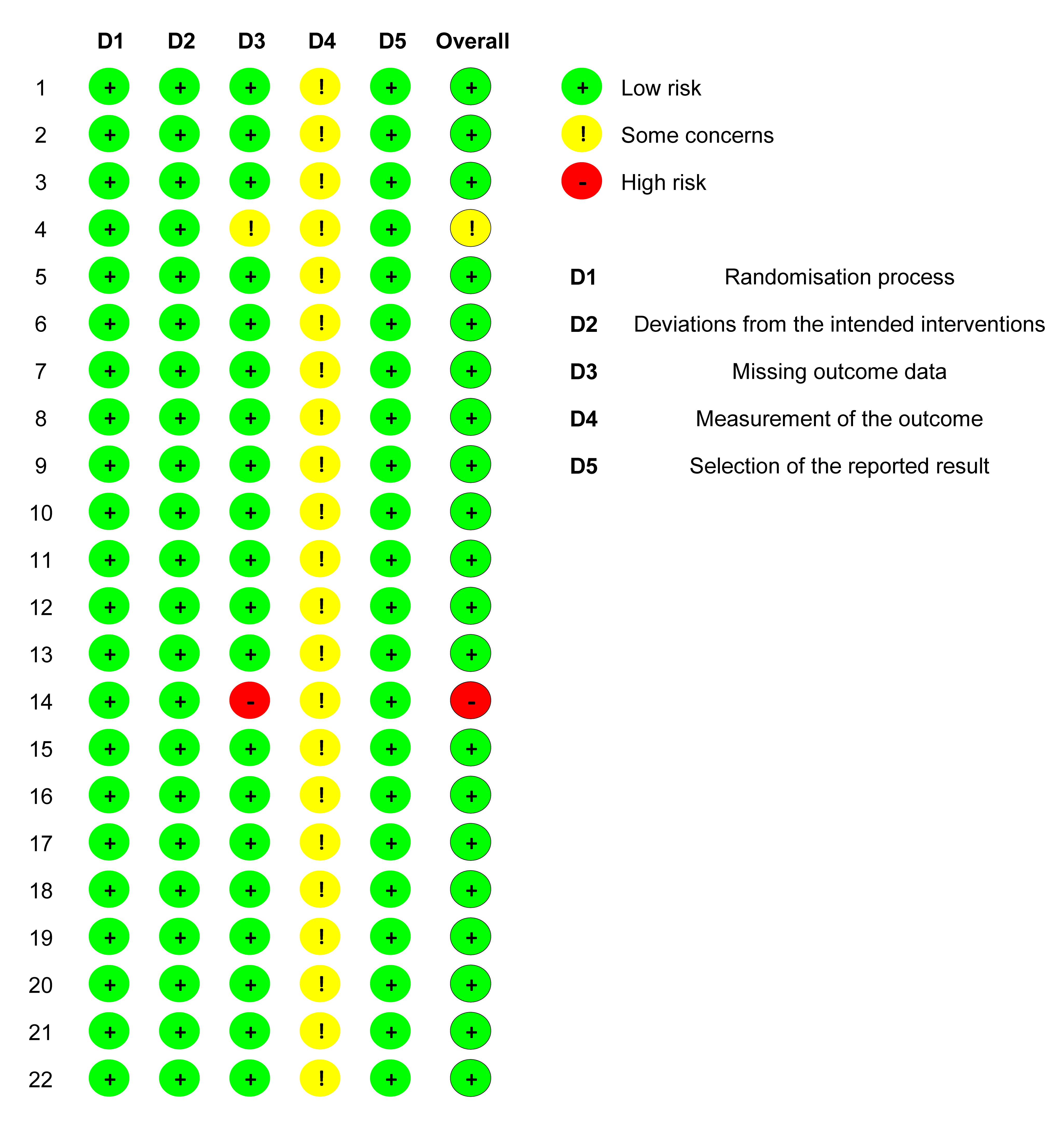


**Supplementary Figure 1.** This is the specific methodological quality evaluation in every domain of each study using Cochrane Risk of Bias Tool (RoB 2.0).





**Supplementary Figure 2.** Funnel plot showing the valuate of publication bias of studies reporting on craving in people with SUD after light, moderate and high intensity exercise intervention. SE, standard error; SMD, standard mean difference.





**Supplementary Figure 3.** Funnel plot showing the valuate of publication bias of studies reporting on depression in people with SUD after light, moderate and high intensity exercise intervention. SE, standard error; SMD, standard mean difference.





**Supplementary Figure 4.** Funnel plot showing the valuate of publication bias of studies reporting on anxiety in people with SUD after light, moderate and high intensity exercise intervention. SE, standard error; SMD, standard mean difference.





**Supplementary Figure 5.** Funnel plot showing the valuate of publication bias of studies reporting on stress in people with SUD after light, moderate and high intensity exercise intervention. SE, standard error; SMD, standard mean difference.





**Supplementary Figure 6.** Funnel plot showing the valuate of publication bias of studies reporting on irritableness in people with SUD after light, moderate and high intensity exercise intervention. SE, standard error; SMD, standard mean difference.





**Supplementary Figure 7.** Funnel plot showing the valuate of publication bias of studies reporting on syndrome in people with SUD after light, moderate and high intensity exercise intervention. SE, standard error; SMD, standard mean difference.



**Supplementary Figure 8.** Funnel plot showing the valuate of publication bias of studies reporting on restless in people with SUD after light, moderate and high intensity exercise intervention. SE, standard error; SMD, standard mean difference.
